# Development of a Home-Based Light Therapy for Fatigue Following Traumatic Brain Injury: Two Case Studies

**DOI:** 10.3389/fneur.2021.651498

**Published:** 2021-09-13

**Authors:** Laura J. Connolly, Jennie L. Ponsford, Shantha M. W. Rajaratnam, Steven W. Lockley

**Affiliations:** ^1^Monash Epworth Rehabilitation Research Centre, Epworth Healthcare, Melbourne, VIC, Australia; ^2^Turner Institute for Brain and Mental Health, School of Psychological Sciences, Monash University, Melbourne, VIC, Australia; ^3^Division of Sleep and Circadian Disorders, Departments of Medicine and Neurology, Brigham and Women's Hospital, Boston, MA, United States; ^4^Division of Sleep Medicine, Harvard Medical School, Boston, MA, United States

**Keywords:** traumatic brain injury, stroke, light therapy, fatigue, sleepiness, sleep disturbance, blue light, melanopsin

## Abstract

**Background and Objectives:** Fatigue and sleep disturbance negatively impact quality of life following brain injury and there are no established treatments. Building on research showing efficacy of blue light therapy delivered via a lightbox in reducing fatigue and daytime sleepiness after traumatic brain injury (TBI), this paper describes the development and implementation of a novel in-home light therapy to alleviate fatigue and sleep disturbance in two case studies.

**Methods:** During the 8-week lighting intervention, participants' home lighting was adjusted to provide high intensity, blue-enriched (high melanopic) light all day as a stimulant and dimmer, blue-depleted (low melanopic) light for 3 h before sleep as a soporific. The sham 8-week control condition resembled participants' usual (baseline) lighting conditions (3,000–4,000 K all day). Lighting conditions were crossed-over. Outcomes were measures of fatigue, subjective daytime sleepiness, sleep quality, insomnia symptoms, psychomotor vigilance and mood. Case study participants were a 35-year old male (5 years post-TBI), and a 46-year-old female (22 years post-TBI).

**Results:** The relative proportion of melanopic lux was greater in Treatment lighting than Control during daytime, and lower during evenings. Participants found treatment to be feasible to implement, and was well-tolerated with no serious side effects noted. Self-reported compliance was >70%. Both cases demonstrated reduced fatigue, sleep disturbance and insomnia symptoms during the treatment lighting intervention. Case 2 additionally showed reductions in daytime sleepiness and depressive symptoms. As expected, symptoms trended toward baseline levels during the control condition.

**Discussion:** Treatment was positively received and compliance rates were high, with no problematic side-effects. Participants expressed interest in continuing the ambient light therapy in their daily lives.

**Conclusions:** These cases studies demonstrate the acceptability and feasibility of implementing a personalized in-home dynamic light treatment for TBI patients, with evidence for efficacy in reducing fatigue and sleep disturbance.

**Clinical Trial Registration:**www.anzctr.org.au, identifier: ACTRN12617000866303.

## Background

Fatigue is the most common and persistent complaint following TBI, with prevalence ranging from 32 to 73% in both early and late recovery stages (1–3). Fatigue imposes significant limitations on physical and social/leisure activities (4) and participation in work and/or study, resulting in poorer quality of life after TBI (3, 5). Sleep disturbance is also commonly reported after TBI, in 30–70% of cases, with frequent disturbances including excessive daytime sleepiness (EDS) manifested as tiredness or drowsiness after insufficient sleep or sleep disruption, as well as hypersomnia, insomnia, reduced sleep efficiency, changes to sleep timing reflecting circadian rhythm changes and sleep apnea (6–8). Unfortunately, no treatments for fatigue or sleep disturbance following brain injury have been shown to be highly effective or to demonstrate lasting improvements. Developing a safe and effective non-pharmacological, non-invasive, and accessible intervention for post-traumatic brain injury fatigue and sleep disturbance is critical given their disabling consequences for these populations (5).

A relatively novel approach to address these complaints is light therapy, which has largely been used to address circadian rhythm disturbance (9) and depressive symptoms associated with seasonal affective disorder (10, 11). In addition to permitting vision, ocular light exposure elicits a range of circadian, neuroendocrine and neurobehavioral responses (12). These “non-visual” responses to light include resetting the circadian pacemaker (13), acute alerting effects (12, 14) and mood enhancement (15). We previously conducted a pilot randomized, placebo-controlled trial of 45 min morning exposure to a light box projecting narrowband short wavelength (blue) light (λ_max_ = 465 nm, 84.8 μW/cm^2^, 39.5 lux, 1.74 × 10^14^ photons/cm^2^/s) in 30 TBI individuals with self-reported fatigue and/or sleep disturbance compared with a narrowband yellow light (control) (λ_max_ = 574 nm, 18.5 μW/cm^2^, 68 lux, 1.21 × 10^12^ photons/cm^2^/s) and no treatment. Exposure to the blue light resulted in significantly reduced fatigue and daytime sleepiness during the 4-week treatment duration, which was not observed in the control or no treatment conditions, with return to almost baseline levels after treatment cessation. There was no significant treatment effect observed for self-reported depression or psychomotor vigilance performance, although there were trends suggestive of potential benefit. The findings of this study suggested that blue light therapy was effective in alleviating fatigue and daytime sleepiness following TBI (16). Similar findings have since been obtained in several other trials in patients with mild (17, 18) and severe TBI (19). On the basis of these trials, a recent systematic review and meta-analysis concluded there was moderate-quality evidence for blue-wavelength light therapy in treating post-TBI depression and fatigue (20). The requirement to sit in front of a light box each morning is somewhat burdensome, however, potentially limiting long-term uptake of this therapeutic option. Alternatively, psychological treatments such as cognitive behavioral therapy have shown promise in treating post-TBI fatigue and sleep disturbance (21, 22), although these studies have been underpowered, and psychotherapy may not be the preferred option for some individuals, and may present challenges for individuals with poor self-awareness (23).

A possible solution is to provide a home-based lighting intervention by regulating ambient lighting which is incorporated seamlessly into the user's environment. A recent inpatient study in stroke patients in a rehabilitation setting found that exposure to naturalistic light emulating a sunlight spectrum amongst stroke patients in a rehabilitation unit resulted in significantly reduced fatigue at discharge compared to a control condition with standard indoor lighting (24). There was no impact on sleepiness or subjective sleep quality, however.

There have been no studies utilizing a home-based lighting intervention approach in patients with acquired brain injury. Changing the ambient lighting environment avoids the burden of daily morning therapy at a fixed time or location and may also increase the duration of participants' exposure to stimulating lighting across the day, which may result in larger and more sustained benefits for fatigue and sleepiness. This is particularly important as individuals with brain injury may experience reduced average light exposure, due to spending extended periods in the home and not participating actively in the community (25, 26). This more holistic approach also offers the advantage of changing evening light, in the hours before sleep, which may have added benefits for sleep (27–29).

In order to address this research gap, we developed a lighting intervention involving exposure to a home-based dynamic light therapy, in which treatment consisted of ambient exposure to blue-enriched white light (CCT >5,000 K) during the daytime and blue-depleted white light (<3,000 K) for 3 h prior to sleep (30). This intervention was compared with control lighting, which consisted of lighting that simulated participants' usual lighting (3,000–4,000 K during the day and evening). Outcomes assessed were fatigue (primary outcome), daytime sleepiness, sleep disturbance, insomnia symptoms, psychomotor vigilance, mood and community participation levels. This paper describes the development and implementation of the home-based ambient lighting intervention, providing an in-depth description of the personalized intervention methodology, and examining its feasibility and acceptability and responses on various measures in two case studies. The results of a pilot randomized-controlled trial are found in a separate paper (30).

## Methods

### Development of the Light Therapy Treatment

#### Lighting Assessment

Participants' current lighting was assessed prior to study commencement to enable researchers to install the appropriate lighting. The Colormunki Light Meter (X-Rite, Grand Rapids, MI, USA) was used to measure participants' home lighting conditions (specific spot measurements at a fixed height in vertical and horizontal planes) and data analyzed using f.luxometer software (f.lux, Los Angeles, CA, USA). Lighting was described and compared for both visual (photopic lux) and non-visual [melanopic Equivalent Daylight Illuminance (EDI) lux] parameters per the CIE Standard International units for ipRGC influenced responses to light (CIE S 026/E:2018) (31). In addition, the Daylight Equivalent Ratio (DER) expresses melanopic EDI as a function of photopic illuminance and is a shorthand for the relative difference in the light spectrum; Melanopic DER values closer to or above 1 represent greater melanopsin stimulation. “Day” measurements were taken with day lighting switched on and exposure to natural light via windows if present. Day measurements therefore represent maximum possible exposure to lighting during daytime. “Evening” measurements were taken with evening lighting switched on and window blinds closed, to approximate night time lighting conditions. A home-lighting questionnaire was used to assess the individual lighting and layout requirements for each participant's home. Priority was given to rooms in which the participant spent the most time.

#### Lighting Intervention

The lighting intervention had two components. Daytime lighting consisted of blue-enriched higher-intensity white light with a correlated color temperature (CCT) of ~ >5,000 K, which participants were instructed to use from waketime and throughout the day. For 3 h prior to sleep in the evening, participants were instructed to use lower intensity and blue-depleted white light (<3,000 K). The goal was to modify lighting to have higher melanopic EDI and DER values during daytime, and lower melanopic EDI and DER values in the evening, as compared to participants' baseline lighting, which was mimicked in the control condition. Participants were asked to maintain a stable light schedule as much as possible day-to-day. Participants provided an estimate of average sleep and wake times in interview at baseline assessment. The start of the evening light exposure was scheduled 3 h before participants' typical sleep time and fixed at that clock time throughout the study. The specific lighting fixtures and lamps used were selected to integrate with participants' existing lighting arrangements. A qualified electrician fitted lighting and bulbs in participants' homes.

For the following two case studies, a combination approach to the lighting intervention was used. Where possible, automated tunable lights, programmed to change the lighting automatically at the right time of day, were installed. Where this was not possible, two types of fixed spectrum lighting, using the concept of “day” and “evening” light, were used. In the instance where there were two circuits in a room, one was fitted with melanopic-enriched light and designated for day time use (e.g., ceiling lights) and another was fitted with dimmer, melanopic-depleted light for use in the evening (e.g., table lamp). Bedside lamps were provided to both participants to facilitate this approach during the Treatment condition. Participants were educated on how to use and time the lights for each condition. In the control condition, the lights were changed as per the Treatment condition, but they were not different in correlated color temperature from participants' normal lighting (typically 3,000–4,000 K). Participants were blinded from the study conditions, and were told that two treatments were being investigated. Floor plans for the two cases are found in Supplementary Figures 1A,B. Treatment protocols can be found in Supplementary Tables 1, 4.

#### Design

The protocol was 5.5 months in length, with a baseline of 2 weeks, followed by two 2-month intervention conditions (Treatment and Control), and a 1-month follow-up. There was no wash-out period between conditions. Both case study participants were allocated to the Treatment-Control sequence. Assessment with outcome measures occurred at baseline, mid- and end-points of Treatment and Control conditions, and at 1-month follow-up. Participants completed the study prior to the COVID-19 pandemic.

#### Participants

The study was approved by the human research ethics committees at Epworth HealthCare and Monash University. Participants provided written informed consent. There was no compensation provided for participation.

The two case studies reported in this paper were identified via a TBI longitudinal follow-up study. They met eligibility criteria: (a) mild-severe TBI at least 3 months earlier; (b) living in the community; (c) self-reporting significant fatigue (Fatigue Severity Scale ≥ 4); no other medical illness accounting for fatigue, pre-injury sleep disorders or chronic fatigue syndrome; (c) no visual impairments that may affect sensitivity and response to light; (d) no transmeridian travel within the preceding 6 weeks; (e) no current use of prescribed and over-the-counter sleep medications; (f) ability to give informed consent as assessed by the referring clinician or recruiting neuropsychologist.

### Outcome Measures

The primary outcome measure was the Brief Fatigue Inventory (BFI) (32), completed at each of the baseline, mid- and end- condition, and follow-up assessments. This was selected as the primary outcome measure due to its suitability in assessing state-like fatigue over the past 24 h, and sensitivity to change observed in previous clinical trials with individuals with TBI (22). Secondary outcomes included the Fatigue Severity Scale (FSS) (33), Epworth Sleepiness Scale (ESS) (34), Pittsburgh Sleep Quality Index (PSQI) (35), Insomnia Severity Index (ISI) (36), Hospital Anxiety and Depression Scale (HADS) (37), Participation Objective Participation Subjective (POPS) (38), and a side effects questionnaire, which were completed at the same time as the BFI. The FSS was included as a secondary fatigue measure as this demonstrated change in a previous light therapy study in TBI (16). Participants also completed a 10-min Psychomotor Vigilance Task (PVT) (39) once during the daytime at each assessment point. Throughout the study, participants completed a daily sleep log and wore wrist actigraphs on the non-dominant wrist (Actiwatch-2, Actiwatch Spectrum or Actiwatch Spectrum Plus; Philips Respironics, Bend, OR, USA), to assess actigraphic sleep parameters. Finally, an “End of Light Therapy Questionnaire” was completed at follow-up, to capture participants' qualitative experiences of the lighting interventions and subjective changes in symptoms (see Supplemental Materials for more details of the outcomes).

## Implementation of the Ambient Light Therapy: Case Studies

### Case Study 1

#### Participant Details and Injury Characteristics

Case 1 was a 35-year-old male who sustained a TBI 5 years earlier in a road traffic accident. He sustained a severe injury, with a Glasgow Coma Scale (GCS) of 3 and a post-traumatic amnesia (PTA) duration of 80 days. A CT scan revealed a left frontal subdural hematoma, but no skull fracture. He also sustained moderate spinal, chest, abdominal and limb injuries, and minor facial injuries. He underwent orthopedic surgery. Duration of acute hospital stay was 34 days. He was not taking any medications during the study. He had no reported history of sleep apnea, other pre-injury sleep disorders or visual impairments. He had completed Year 11 of high school. At the time of study enrollment he was working full time in an office and living with his partner. He self-reported significant fatigue at initial screening (FSS = 6.00).

#### Development of Treatment Protocol

Supplementary Figure 1A shows a floor plan for Case 1's home. He reported spending a lot of time in his study playing computer games (day and night, often until bedtime; position E). He reported waking at 7–8 a.m. weekdays and 11 a.m. weekends, and sitting in the study for breakfast prior to leaving for work. He showered during evenings, between 8 and 10 p.m. His usual sleep time was 11 p.m. weekdays and 2 a.m. weekends.

During the treatment condition, spectrum switching (tunable) globes with both low and high melanopic light (Scene Switch, Philips Electronics Australia Limited, NSW, Australia) were utilized throughout the apartment in the ceiling lights (living room – position A, kitchen – position B, bathroom – position C, study – position E, bedroom – position F), based on use in both daytime and evenings. Furthermore, as Case 1 spent a lot of time in his study (position E), an additional desk lamp was provided during the Treatment condition, in order to maximize light exposure during daytime and provide an alternative source of appropriate low CCT light during evenings. f.lux software (40) was used to automatically adjust the color temperature of both his computer display and smart phone to reduce exposure to high melanopic light during evenings. As Case 1 reported typically sleeping at 11 p.m., and a desire to maintain this on weekends, 8 p.m. was the time selected to transition from daytime to evening lighting. Case 1's partner was educated about the lighting and reported compliance with the treatment protocol. Control lighting was chosen to approximate Baseline lighting. A summary of the lighting at baseline and that installed for the Treatment and Control conditions can be found in Supplementary Table 1. A summary of the lighting parameters for position and study condition can be found in Supplementary Table 2 (Baseline), Table 1 (primary Treatment and Control measures), and Supplementary Table 3 (additional Treatment and Control measures). Case 1 commenced the study in Spring and completed in Autumn.

**Table 1:** Case 1 Treatment and Control condition lighting measurements.

**Location (Time)**	**Height, plane**	**Pos**.	**Photopic lux** **TREAT**.	**Photopic lux** **CONTROL**.	**CCT (K)** **TREAT**.	**CCT (K)** **CONTROL**.	**Melanopic a-opic EDI (lux)** **TREAT**.	**Melanopic a-opic EDI (lux)** **CONTROL**.	**Melanopic DER** **TREAT**.	**Melanopic DER** **CONTROL**.
			**Mean ±** **SD**	**Mean ±** **SD**	**Mean ±** **SD**	**Mean ±** **SD**	**Mean ±** **SD**	**Mean ±** **SD**	**Mean ±** **SD**	**Mean ±** **SD**
Living area (Day)	52'', vert x 4	A	100.90 ± 66.87	130.09 ± 74.85	5,605 ± 364	4,592 ± 751	88.54 ± 60.25	106.23 ± 77.11	0.87 ± 0.03	0.78 ± 0.11
	72'', horiz	A	Missing	303.25	Missing	4,023	Missing	200.50	Missing	0.66
Living area (Evening)	52'', vert x 4	A	28.37 ± 21.20	34.82 ± 20.43	2,836 ± 111	3,728 ± 204	12.65 ± 10.00	22.02 ± 14.16	0.44 ± 0.02	0.62 ± 0.04
	72'', horiz	A	167.16	185.97	2,995	3,802	75.44	112.66	0.45	0.61
Kitchen (Day)	52'', vert x 4	B	103.98 ± 78.39	192.52 ± 189.91	5,343 ± 292	4,904 ± 1,946	91.85 ± 75.82	183.39 ± 224.75	0.86 ± 0.05	0.80 ± 0.22
	72'', horiz	B	182.14	289.49	5,841	3,077	160.8	150.54	0.88	0.52
Kitchen (Evening)	52'', vert x 4	B	69.07 ± 40.14	130.92 ± 107.62	3,482 ± 867	4,666 ± 2,230	44.51 ± 40.47	118.58 ± 136.76	0.58 ± 0.17	0.75 ± 0.25
	72'', horiz	B	171.02	343.24	3,137	2,849	84.46	154.88	0.49	0.45
Bathroom (Day)	52'', vert x 4	C	147.40 ± 103.61	203.14 ± 131.15	5,032 ± 452	5,602 ± 1,552	120.92 ± 94.37	198.35 ± 155.88	0.79 ± 0.06	0.91 ± 0.15
	72'', horiz	C	333.71	226.41	5,560	3,354	272.65	133.36	0.82	0.59
Bathroom (Evening)	52'', vert x 4	C	No blinds in bathroom.^a^							
	72'', horiz	C								
Study (Day)	52'', vert x 4	E	118.12 ± 73.84	145.76 ± 133.88	5,386 ± 402	4,512 ± 1,267	100.03 ± 67.58	124.30 ± 141.52	0.83 ± 0.05	0.75 ± 0.15
	72'', horiz	E	354.08	440.37	5,858	3,308	303.58	244.31	0.86	0.55
Study (Evening)	52'', vert x 4	E	72.22 ± 36.35	27.01 ± 4.74	2,838 ± 88	2,793 ± 79	30.66 ± 16.26	11.85 ± 2.40	0.42 ± 0.02	0.44 ± 0.02
	72'', horiz	E	221.18	288.62	2,936	2,966	95.94	135.04	0.43	0.47
Bedroom (Day)	52'', vert x 4	F	68.74 ± 43.53	235.54 ± 161.86	5,408 ± 373	5,458 ± 1,027	59.90 ± 43.10	220.64 ± 176.84	0.87 ± 0.05	0.88 ± 0.12
	72'', horiz	F	264.36	355.77	5,970	4,526	228.21	267.49	0.86	0.75
Bedroom (Evening)	52'', vert x 4	F	23.38 ± 6.59	60.00 ± 30.10	2,785 ± 102	3,051 ± 342	9.58 ± 2.70	29.44 ± 11.64	0.41 ± 0.02	0.51 ± 0.06
	72'', horiz	F	241.06	223.10	2,972	3,566	105.22	128.30	0.44	0.58
Bedroom (Evening; lamp only)^b^	52'', vert x 4	F	22.31 ± 20.98		2,153 ± 126		6.43 ± 5.72		0.30 ± 0.05	
	72'', horiz	F	28.38		2,164		8.36		0.29	

*CCT, correlated color temperature; EDI, Equivalent Daylight Illuminance; DER, Daylight Equivalent Ratio; Pos., Position. Only the melDER is shown here; DER values for the other photoreceptors, found in Supplementary Materials, can be calculated by dividing the α-opic EDI value by the photopic lux provided*.

*The aim of the intervention was to increase the melEDI and DER during the day and decrease them during the evening during the Treatment compared to the Control intervention. This was typically associated with a daytime increase and evening decrease in CCT during the Treatment*.

*Average calculations found in text generated using 72'' measurements*.

a *As evening light measurements were taken with window blinds closed, to approximate night time lighting conditions, measurements were not possible in locations without blinds*.

b *An additional measure that was not captured in the Control condition*.

#### Outcomes

The photopic lux, melanopic EDI and melanopic DER for the Treatment and Control conditions are found in Tables 1, 2, respectively. They show that Treatment consisted of melanopic-enriched lighting, with a higher melanopic EDI lux (*M* = 241.31, *SD* = 61.95; measured in the horizontal plane at 72”) and DER (*M* = 0.86, *SD* = 0.03) during daytime, as compared to Control lighting (melanopic EDI *M* = 199.24, *SD* = 57.88; melanopic DER *M* = 0.62, *SD* = 0.09). In the evening, the Treatment had lower melanopic EDI lux (*M* = 73.88, *SD* = 38.33) and DER (*M* = 0.42, *SD* = 0.08) compared to Control light (melanopic EDI *M* = 132.72, *SD* = 17.50; DER *M* = 0.52, *SD* = 0.08). General illuminance (photopic lux) was not greater in daytime Treatment lighting (*M* = 283.57, *SD* = 77.77) relative to Control (*M* = 323.06, *SD* = 80.14) which illustrates that the relative proportion of melanopic lux can be increased whilst still maintaining the same visual illuminance. Treatment was, however, lower in photopic lux (*M* = 165.76, *SD* = 83.13) than Control (*M* = 260.23, *SD* = 69.74) during evenings.

**Table 2:** Case 1 and 2 outcomes at baseline, mid- and end Treatment and mid- and end Control.

**Outcome**	**Case**	**Baseline**	**Mid treatment**	**End treatment**	**Mid control**	**End control**	**Follow up**
BFI	1	5.67	1.56	1.00	6.78	6.33	7.00
	2	4.33	5.89	2.33	2.78	3.89	4.56
ESS	1	3.00	1.00	2.00	1.00	1.00	0.00
	2	11.00	6.00	7.00	6.00	11.00	7.00
FSS	1	5.22	2.22	2.11	3.67	5.78	5.67
	2	2.33	3.56	2.00	2.11	3.11	3.33
HADS Depression	1	5.00	2.00	3.00	4.00	2.00	5.00
	2	6.00	3.00	3.00	4.00	4.00	6.00
ISI	1	18.00	2.00	7.00	11.00	20.00	8.00
	2	15.00	17.00	13.00	13.00	15.00	11.00
MEQ	1	34.00	49.00	42.00	38.00	39.00	41.00
	2	72.00	74.00	70.00	73.00	72.00	70.00
PSQI Global	1	8.00	3.00	2.00	5.00	5.00	3.00
	2	11.00	7.00	8.00	8.00	8.00	9.00
PVT Mean RT (ms)	1	344.44	–	–	–	–	–
	2	310.44	300.03	314.75	288.65	268.51	–
PVT Fastest 10% RT (ms)	1	281.63	–	–	–	–	–
	2	237.02	238.14	223.33	233.77	217.24	–
POPS (Objective)	1	−0.25	0.10	−0.13	0.96	−0.12	0.40
	2	1.06	1.58	0.92	0.09	0.51	0.50
Sleep Onset^a^^c^	1	22:43	00:48	–	–	00:14	01:09
	2	21:39	–	–	20:52	21:42	21:37
Sleep Offset^a^^c^	1	06:45	07:04	–	–	08:23	07:59
	2	05:50	–	–	03:41	05:25	04:34
SOL^b^^c^	1	10.59	60.90	–	–	6.69	7.17
	2	5.11	–	–	8.00	7.94	10.21
WASO^b^^c^	1	46.53	11.97		–	21.78	19.36
	2	69.31	–	–	69.87	73.41	68.50
TST^b^^c^	1	435.48	364.00	–	–	468.36	470.42
	2	421.85	–	–	339.60	389.35	347.86
Sleep efficiency (%)^c^	1	87.45	74.68	–	–	90.89	90.78
	2	83.74	–	–	75.76	81.27	79.09

*BFI, Brief Fatigue Inventory; ESS, Epworth Sleepiness Scale; FSS, Fatigue Severity Scale; HADS, Hospital Anxiety and Depression Scale; ISI, Insomnia Severity Index; MEQ, Morningness Eveningness Questionnaire; PSQI, Pittsburgh Sleep Quality Index; POPS, Participation Objective Participation Subjective; PVT, Psychomotor Vigilance Task; SOL, Sleep onset latency; TST, Total sleep time; WASO, Wake after sleep onset. Some outcomes missing due to equipment failure*.

a *Values represent real clock times, in 24 h time*.

b *Outcome is in minutes*.

c *Individual actigraphic sleep episodes were inspected and aligned with sleep diaries (see Supplementary Materials). Sleep outcome is derived from the average of data in mid- to end-treatment condition periods*.

He was randomly allocated to sequence Treatment-Placebo. Table 2 summarizes his baseline scores for the study assessment measures, in addition to mid- and end of Treatment and Control conditions.

**Figure 1:**
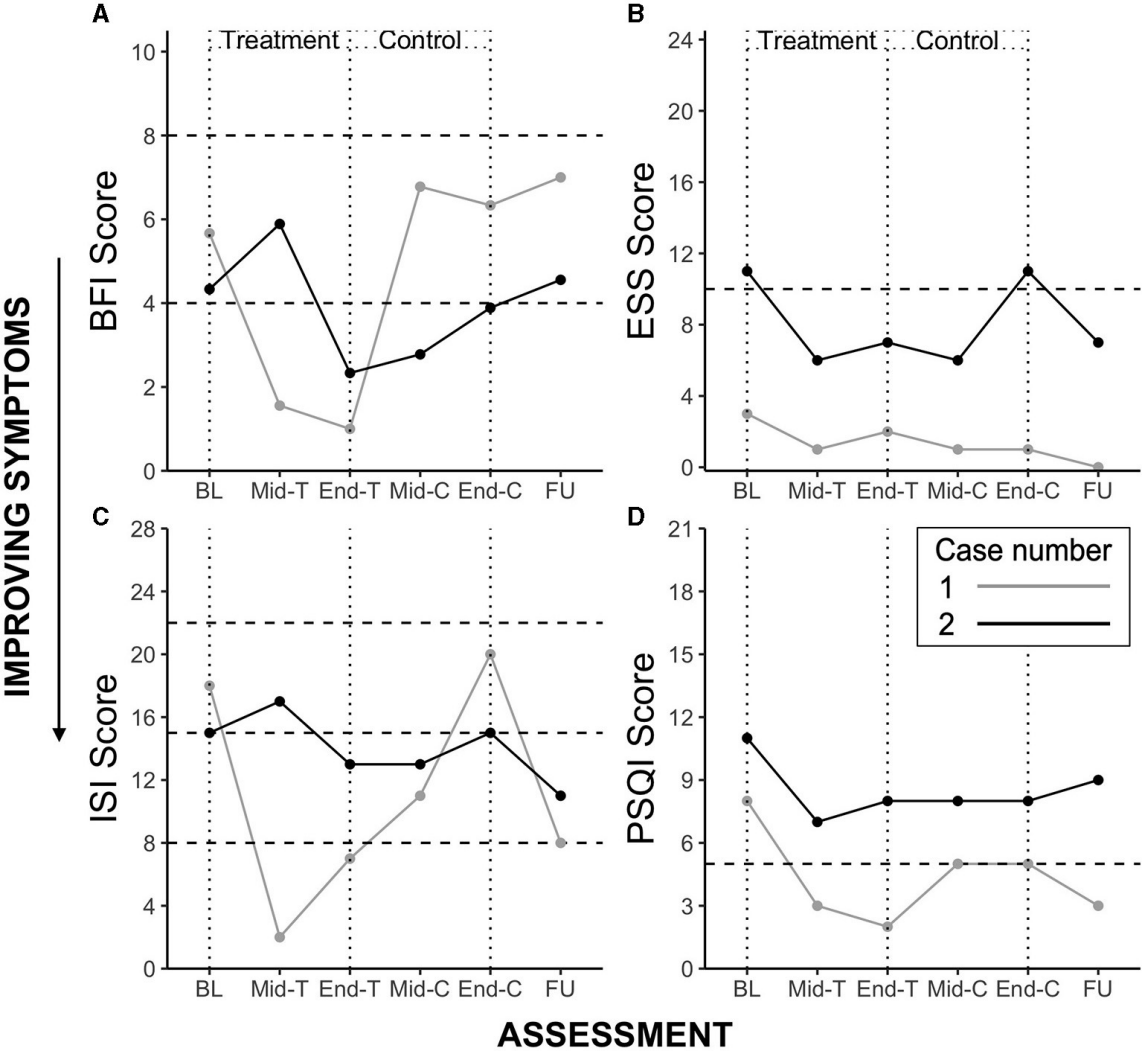
Case study 1 and 2 outcome scores for Brief Fatigue Inventory (BFI) **(A)**, Epworth Sleepiness Scale (ESS) **(B)**, Insomnia Severity Index (ISI) **(C)**, and Pittsburgh Sleep Quality Index (PSQI) **(D)**. BL, Baseline; T, Treatment; C, Control; FU, Follow Up. Horizontal dotted lines represent clinically significant cut-offs. BFI: range 0–10, scores 4–7 represent moderate fatigue and suggest a need for intervention, scores ≥ 8 represent severe fatigue; ESS: range 0–24, score > 10 suggests clinically significant daytime sleepiness; ISI: range 0–28, a score of 8–14 indicates subthreshold insomnia, 15–21 clinically moderate, and 22 or greater severe clinical insomnia; PSQI, range 0–21, scores ≥ 5 indicate clinically significant sleep disturbance.

The participant demonstrated marked reductions in fatigue (both on the BFI and FSS), depressive symptoms (below clinically significant threshold at Baseline), insomnia severity and improvements in sleep quality. Clinically significant reductions (i.e., reducing below the clinically significant threshold during or at the end of the Treatment period, compared to Baseline) were observed from Baseline to mid- and end Treatment assessment points in fatigue (−4.67), insomnia symptoms (−11.00) and sleep disturbance (−6.00). Unfortunately mid-to-end of treatment actigraphy data were missing due to equipment failure, however there was a significant reduction in wake after sleep onset observed at the mid-treatment point (11.97 vs. 46.53 min. during the Baseline period), which increased at Control and Follow Up periods, although this was accompanied by increases in sleep onset latency, and reductions in total sleep time and sleep efficiency. Symptoms typically increased during the Control condition. These trends are observable in the Figure 1 for the four main outcomes.

In terms of side effects, Case 1 reported moderate visual problems, mild sleep, memory and concentration difficulties, fatigue and irritability during the Treatment condition. He observed these symptoms to be lesser than or the same as usual, aside from rating a slight increase in visual disturbance. During the Control condition he reported moderate eye irritation, visual problems, sleep problems, and fatigue, and mild memory and concentration difficulties, drowsiness and irritability.

In the End of Study Questionnaire he rated a “considerable improvement” in fatigue, sleep, participation in daily life and a “slight improvement” in mood and quality of life during the Treatment condition. He found all of these domains to worsen during the Control condition. He also reported increased productivity during the Treatment condition, and that his more alert state was also noted by his work colleague. He reported 70% compliance with the Treatment conditions, in terms of his ability to use treatment lighting, and transition from day to evening light at the designated hour, and found his only issue to be an occasional delay in changing light settings at the time agreed. In terms of treatment feasibility, he found the intervention to be simple to implement. He remarked that “evening” lighting was relaxing, and reported “very high” satisfaction with the treatment and a desire to incorporate light therapy in his home following the completion of the study.

### Case Study 2

#### Participant Details and Injury Characteristics

Case 2 was a 46-year-old female who sustained a TBI 22 years earlier in a motorcycle accident. She sustained a severe injury, with a GCS of 9 and a PTA duration of 51 days. A CT scan revealed a right frontal sinus fracture. She also sustained major spinal injury. Case 2 additionally experienced generalized seizures in the first week following injury. Duration of acute hospital stay was 44 days prior to a 12-month inpatient rehabilitation stay. In terms of medications, she was taking paracetamol, as needed for pain, meloxicam (pain), and albuterol (asthma), during the study. She had no reported history of sleep apnea, other pre-injury sleep disorders or visual impairments. She had completed Year 12 of high school. At the time of study enrollment she was working part-time in an outdoor setting, and living in a self-contained unit attached to her parents' house. She self-reported significant fatigue at initial screening (FSS = 4.89).

#### Development of Treatment Protocol

Supplementary Figure 1B shows the home floor plan for Case 2. She reported using her iPad most evenings in the living room (position B), showering in the evenings, and using the bathroom multiple times during the night (position D). Her usual sleep time was 10 p.m. and wake time 5 a.m.

During the Treatment condition, a spectrum switching globe with both low and high melanopic light (Scene Switch, Philips Electronics Australia Limited, NSW, Australia) was utilized in the living ceiling light (position B), based on daytime and evening use. Living room lighting was supplemented by a lamp with low melanopic light (GoodNight LED bulb, Lighting Science, RI, USA), situated where Case 2 would typically sit during evenings. Low melanopic light (GoodNight LED bulb, Lighting Science, RI, USA) was also used as the primary lighting in the bedroom ceiling light, due to primary evening use. Additionally, a tunable lamp was used at the bedside, which adjusted to melanopic-enriched or depleted light based on time of day (position A; Genesis DynaSpectrum HealthE LED Lamp, Lighting Science, RI, USA). Based on exclusively daytime use, high melanopic light was installed in the kitchen (position C; GoodDay LED bulb, Lighting Science, RI, USA). As options for lighting were limited in the bathroom (fluorescent tube; position D), and on the basis of late and nighttime use of this space, low melanopic light was used (Osram 60 cm T8 fluorescent tube, 3,000 K, 1,350 lm, 18 W, Munich, Germany). “Night Shift” was used to automatically adjust the color temperature of her iPad display, to reduce exposure to high melanopic light during evenings. As Case 2 reported typically sleeping at 10 p.m., 7 p.m. was the time selected to transition from daytime to evening lighting. Control lighting was chosen to approximate Baseline lighting. A summary of the lighting at baseline and that installed for the Treatment and Control conditions can be found in Supplementary Table 4. A summary of the lighting parameters for photopic lux, irradiance and CCT values, plus α-opic EDI, and melanopic DER values across room and study condition can be found in Supplementary Table 5 (Baseline), Table 3 (primary Treatment and Control measures) for measures in the horizontal plane at a height of 72” and the average of four measures 90 degrees apart in the vertical plane at a height of 54”. Additional light measures for Treatment and Control can be found in Supplementary Table 6. Case 2 commenced the study in Autumn and completed in Spring.

**Table 3:** Case 2 Treatment and Control condition lighting measurements.

**Location (Time)**	**Height, plane**	**Pos**.	**Photopic lux TREAT**.	**Photopic lux** **CONTROL**	**CCT (K)** **TREAT**.	**CCT (K)** **CONTROL**	**Melanopic a-opic EDI (lux)** **TREAT**.	**Melanopic a-opic EDI (lux)** **CONTROL**	**Melanopic DER** **TREAT**.	**Melanopic DER** **CONTROL**
			**Mean ±** **SD**	**Mean ±** **SD**	**Mean ±** **SD**	**Mean ±** **SD**	**Mean ±** **SD**	**Mean ±** **SD**	**Mean ±** **SD**	**Mean ±** **SD**
Bedroom (Day)	52'', vert x 4	A	27.21 ± 7.71	34.10 ± 27.12	3,377 ± 559	3,626 ± 402	19.34 ± 10.18	19.83 ± 17.73	0.67 ± 0.19	0.55 ± 0.05
	72'', horiz	A	106.47	114.23	2,136	2,500	32.13	45.19	0.30	0.40
Bedroom (Evening)	52'', vert x 4	A	11.69 ± 2.48	27.35 ± 15.79	2,041 ± 35	4,608 ± 305	2.76 ± 0.65	17.75 ± 11.91	0.24 ± 0.01	0.63 ± 0.06
	72'', horiz	A	107.38	101.39	2,021	4,437	25.99	59.60	0.24	0.59
Living (Day)	52'', vert x 4	B	15.30 ± 10.35	64.67 ± 66.37	4,483 ± 329	4,210 ± 216	10.55 ± 7.76	41.61 ± 43.04	0.66 ± 0.05	0.63 ± 0.05
	72'', horiz	B	42.51	37.74	4,899	3,985	30.95	21.69	0.73	0.57
Living (Evening)	52'', vert x 4	B	12.28 ± 8.34^b^	84.22 ± 121.61	4,463 ± 297^b^	4,176 ± 866	8.54 ± 6.10^b^	70.01 ± 106.35	0.68 ± 0.03^b^	0.72 ± 0.14
	72'', horiz	B	25.38	28.13	4,777	3,408	18.10	16.42	0.71	0.58
Kitchen (Day)	52'', vert x 4	C	118.13 ± 99.16	184.96 ± 97.99	4,493 ± 282	4,162 ± 303	85.84 ± 72.59	118.01 ± 68.57	0.71 ± 0.07	0.62 ± 0.06
	72'', horiz	C	83.48	230.94	4,199	3,898	53.47	128.72	0.64	0.56
Kitchen (Evening)	52'', vert x 4	C	62.88 ± 70.13^c^	49.58 ± 49.47	4,299 ± 164^c^	3,712 ± 359	42.56 ± 47.66^c^	25.26 ± 25.12	0.67 ± 0.01^c^	0.52 ± 0.06
	72'', horiz	C	36.12	64.12	4,165	3,897	24.19	32.24	0.67	0.50
Bathroom (Day)	52'', vert x 4	D	40.01 ± 16.72	22.80 ± 26.65	2,955 ± 432	4,841 ± 604	17.41 ± 7.40	19.62 ± 24.74	0.44 ± 0.10	0.80 ± 0.07
	72'', horiz	D	191.64	15.90	2,836	4,642	75.50	11.95	0.39	0.75
Bathroom (Evening)	52'', vert x 4	D	No blinds in bathroom.^a^	56.80 ± 35.49^d^	No blinds in bathroom.^a^	2,744 ± 55^d^	No blinds in bathroom.^a^	21.72 ± 13.65^d^	No blinds in bathroom.^a^	0.38 ± 0.01^d^
	72'', horiz	D		176.31		2,832		68.65		0.39

*CCT, correlated color temperature; EDI, Equivalent Daylight Illuminance; DER, Daylight Equivalent Ratio; Pos., Position. Only the melDER is shown here; DER values for the other photoreceptors, found in Supplementary Materials, can be calculated by dividing the α-opic EDI value by the photopic lux provided*.

*The aim of the intervention was to increase the melEDI and DER during the day and decrease them during the evening during the Treatment compared to the Control intervention. This was typically associated with a daytime increase and evening decrease in CCT during the Treatment*.

*Average calculations found in text generated using 72” measurements. Bedroom and bathroom measurements were excluded from Treatment “day” average calculations as these spaces were used exclusively during evenings and daytime lighting was not fitted. The following measures were excluded from Treatment “evening” calculation: Kitchen measurement excluded as this space was used exclusively during daytime and evening lighting was not fitted; Living was excluded from “evening” calculation as daytime lighting was primarily measured, as noted below; Bathroom was excluded as no blinds were available to block natural light from windows in this space*.

*Bedroom and bathroom measurements were excluded from Control “day” average calculations as these spaces were used exclusively during evenings and daytime lighting was not fitted during Treatment. The following measures were excluded from Control “evening” calculation: Kitchen measurement excluded as this space was used exclusively during daytime and evening lighting was not fitted during Treatment; Living was excluded from “evening” calculation as daytime lighting was primarily measured, as noted below; Bathroom was excluded as no blinds were available to block natural light from windows in this space*.

a *As evening light measurements were taken with window blinds closed, to approximate night time lighting conditions, measurements were not possible in locations without blinds, if assessed during daytime*.

b *Lighting Science GoodNight light (2,175 K, 600 lm) used in a lamp in this space for evening use, with an established M/P of 0.33. It is likely light was blended with overhead daytime lighting at the time of measurement due to researcher error*.

c *Single spectrum high melanopic light only was installed in the kitchen, based on exclusive daytime use. Evening measures are therefore not reflective of actual night time exposure in this location*.

d *Measurement was captured in evening, after sunset*.

#### Outcomes

Table 3 shows the Treatment and Control light conditions, respectively, for Case 2. It shows that Treatment consisted of melanopic-enriched lighting, with a higher melanopic DER (*M* = 0.68, *SD* = 0.06; measured in the horizontal plane at 72”) during daytime, compared to Control light (*M* = 0.57, *SD* = 0.10) (Table 3). This difference was not reflected in the daytime melanopic EDI, however, which was lower (*M* = 41.21, *SD* = 15.93) relative to the Control condition (*M* = 75.21, *SD* = 75.68), nor photopic lux (Treatment *M* = 62.99, *SD* = 28.96; Control *M* = 134.34, *SD* = 136.61). These lack of differences are likely due to the exclusion of the bedroom and bathroom from the Treatment calculation, on the basis of these spaces being used primarily during evenings and designed with exclusively low melanopic light. There was, however, as intended, a reduction in melanopic EDI in Treatment (25.99 in the bedroom) compared to control (59.60 in the bedroom), during evening. There was also a reduction in melanopic DER (0.24 in the bedroom in Treatment vs. 0.59 in the bedroom) during evening.

She was randomly allocated to sequence Treatment-Placebo. Table 2 summarizes her baseline scores for the study assessment measures, in addition to mid- and end of Treatment and Control conditions.

The participant demonstrated marked reductions in fatigue (BFI), daytime sleepiness, depressive symptoms (below clinically significant threshold at Baseline), insomnia symptoms and sleep disturbance during Treatment. Clinically significant reductions were observed from Baseline to mid- and end Treatment assessment points in fatigue (−2.00), daytime sleepiness (−4.00), and insomnia symptoms (−2.00). As in Case 1, symptoms typically increased during the Control condition, except sleep disturbance which remained stable, and increased during follow-up. These trends are observable in Figure 1 for the four main outcomes.

In terms of side effects, Case 2 reported mild abdominal discomfort, sleep problems, memory and concentration difficulties, and fatigue during the Treatment condition. She observed these symptoms to be the same as usual. During the Control condition, she reported mild headache, abdominal discomfort, drowsiness, fatigue, and irritability, and moderate sleep problems and memory and concentration difficulties.

In the End of Study Questionnaire she rated a “considerable improvement” in fatigue and “slight improvement” in sleep, mood, and quality of life during the Treatment condition. She found these domains to worsen during the Control condition, except quality of life which was stable. She also noted that morning treatment light was particularly helpful to get her going for the day, and that evening lighting was “very relaxing.” She reported 80% compliance with the Treatment conditions. The only study challenge was remembering daily completion of paper sleep diaries. She reported she was “mostly satisfied” with the treatment, and wished to incorporate light therapy in her home following the completion of the study.

## Discussion

This study describes the implementation of a novel in-home light therapy to alleviate fatigue and sleep disturbance in two case studies. Previous studies have examined the use of short-duration early morning light box therapy using blue- or blue-enriched light to treat fatigue associated with TBI (16–19) and cancer (41, 42) and found significant reductions in fatigue and daytime sleepiness during the day, but no improvement in insomnia symptoms or sleep quality. Those studies with follow-up assessments found symptoms returned to baseline upon withdrawal of treatment (16, 19). Utilizing light boxes was burdensome for some individuals, and may not result in sustained effect throughout the day. Morning light therapy is also less likely to provide benefit than active evening light intervention. Our protocol was designed to provide light therapy in a more holistic fashion. Embedding light therapy in the ambient light environment has potential to increase treatment efficacy, enhance compliance and reduce patient burden.

Ambient light therapy has been trialed in care homes with geriatric patients, many of whom have dementia, with the aim of improving cognition, sleep and mood in residents, by increasing the intensity (43) and/or the short-wavelength (44–47) content of the light in common areas during the daytime, and have shown promise. Consistent with our overall findings, the results of these studies have shown that changing ambient light conditions can reduce disturbance in sleep; advancing sleep timing (46), increasing total sleep time and efficiency (45), and increasing subjective sleep quality (44, 45). They have also shown significant changes in mood (reducing anxiety and depressive symptoms) (43–46), and behavior; reducing agitation (43–45, 47) and increasing daytime activity levels (46). One study also demonstrated light therapy may attenuate cognitive deterioration in older individuals (43). Most studies, however, only employed intervention lighting during daytime, and did not modify evening lighting. Individuals therefore may have been disrupted by high melanopic light during evenings. Many were also not sham-controlled and have not compared two separate lighting conditions with different spectral properties.

The current study has taken this approach several steps further by: (i) providing ambient light therapy during the day and evening in individuals with TBI living at home rather than in a facility; and (ii) addressing both daytime and evening lighting, with the intent to reduce sleepiness in the daytime and increase sleepiness in evening, to promote sleep. The outcomes of the two case studies of individuals with TBI still experiencing fatigue and sleep disturbance many years after injury showed this intervention to be effective in reducing fatigue, insomnia symptoms and sleep disturbance for these cases, with Case 2 also showing reductions in daytime sleepiness and depressive symptoms. In line with our expectations, symptoms returned to near-baseline levels during the Control condition. Self-rated compliance with Treatment conditions was 70% (Case 1) and 80% (Case 2). Case 1 reported a minor increase in visual disturbance during the Treatment condition but no adverse events requiring discontinuation. Other symptoms reported by Cases 1 and 2 during the Treatment condition were in line with usual symptoms, and with were not significantly different from symptoms reported during the Control condition.

Both participants reported positive experiences during the study in terms of their symptoms and found it to be feasible to implement on a daily basis. Case 1 additionally found his concentration and work productivity to be increased. They were satisfied with the intervention and wished to continue light therapy in their day-to-day life following the cessation of the study. Furthermore, both cases remarked that they found “evening” lighting to be relaxing.

The case studies allowed us to refine our approach to lighting design and selection. Challenges associated with implementing a home-based lighting intervention included variations in light fixtures across homes, which requires knowledge of suitable lighting options for both high and low melanopic light appropriate for those fixtures, and a limited number of available circuits in a given space to provide both day- and evening-appropriate lighting. While tunable LED smart lights can provide the solution, they may require the use of a wi-fi internet connection, which means these lights may not be suitable in homes without wi-fi, as in Case 2. These challenges may also differ between older and newer build homes. Older houses tend to have fewer lighting fixtures which may result in reduced overall illuminance, relative to newer homes, for example.

The challenge in implementing the “same” intervention in different environments is illustrated by comparing the cases. Case 1 was a good example in that we were able to successfully increase melanopic and photopic lux during daytime and decrease these during evening in the Treatment condition. In Case 2, however, while we succeeded in increasing the relative proportion of melanopic light during daytime in Treatment, compared to Control condition, we did not increase overall melanopic illuminance. This case was limited by the number and type of fixtures in the home. In retrospect, selecting higher lumen output as well as higher melanopic EDI lamps for daytime use where there are fewer lamps is warranted. Further consideration should also be given to whether there are ways to increase melanopic EDI illuminance further by providing additional daytime lighting (e.g., floor lamps, strip lights etc.). Further, while the cost of implementing Treatment lighting was approximately $400–600 AUD ($300–450 USD) for each of these cases, this was inclusive of electrician visits, and could be achieved for less in a non-research setting (for example if the patients or their carers changed the light bulbs). There is a trade-off between using fixed spectra lamps in two circuits which are likely less expensive but require multiple circuits (e.g., ceiling and table lamp), or tunable lamps in a single position which will ease compliance but are more expensive. Regardless of which approach is chosen, however, the costs are relatively small in relation to the continual clinical benefits gained and compared to a pharmaceutical treatment, for example. It may be highly cost-effective in the longer run for healthcare funders to provide these home-based lighting solutions as a therapeutic option. Lastly, both cases had the same sequence of study conditions (Treatment-Control). As this study was a case description rather than an evaluation of sequence, the effect of this is unknown.

It needs to be acknowledged that these cases were not controlled studies and no tests of statistical significance were conducted to identify the significance of the individual changes reported. Nevertheless, this approach shows promise as a novel, feasible and individualized approach to light therapy, that makes few cognitive demands for compliance. It needs to be evaluated in a controlled trial involving individuals with TBI and also, potentially, those with stroke who experience post-injury problems with fatigue and/or sleep disturbance.

## Data Availability Statement

The raw data supporting the conclusions of this article will be made available by the authors, without undue reservation.

## Ethics Statement

The studies involving human participants were reviewed and approved by Epworth HealthCare Human Research Ethics Committee and Monash University Human Research Ethics Committee. The patients/participants provided their written informed consent to participate in this study. Written informed consent was obtained from the individual(s) for the publication of any potentially identifiable images or data included in this article.

## Author's Note

This trial was registered with the Australian and New Zealand Clinical Trials Registry, www.anzctr.org.au, ACTRN12617000866303. In addition, a Clinical Trials Notification (CTN) was submitted to the Therapeutic Goods Administration (TGA) prior to commencement of the study.

## Author Contributions

SL, JP, SR, and LC contributed to the conception and design of the study. LC wrote the first draft of the manuscript. SL and JP wrote sections of the manuscript. All authors contributed to manuscript revision, read, and approved the submitted version.

## Funding

This work was supported by funding from The Summer Foundation, Monash University, and Epworth HealthCare. The lighting used in the study was purchased from commercial retailers. The choice of lamps used was based on availability, the appropriate spectrum, cost and compatibility with existing fixtures.

## Conflict of Interest

SR is the Program Leader for the CRC for Alertness, Safety and Productivity, Australia; Director (now Chair) of the Sleep Health Foundation. He has received grants from Vanda Pharmaceuticals, Philips Respironics, Cephalon, Rio Tinto, BHP Billiton and Shell which are not related to this paper. He has received equipment support and consultancy fees through his institution from Optalert, Compumedics, Teva Pharmaceuticals, and Circadian Therapeutics, which are not related to this paper. SL has had a number of commercial interests in the last 3 years (2018–20). His interests were reviewed and managed by Brigham and Women's Hospital and Partners HealthCare in accordance with their conflict of interest policies. No interests are directly related to the research or topic reported in this paper but, in the interests of full disclosure, are outlined below. SL has received consulting fees from the BHP Billiton, EyeJust Inc., Noble Insights, Rec Room, Six Senses, Stantec and Team C Racing; and has current consulting contracts with Akili Interactive; Apex 2100 Ltd.; Consumer Sleep Solutions; Headwaters Inc.; Hintsa Performance AG; KBR Wyle Service, Light Cognitive; Lighting Science Group Corporation/HealthE; Look Optic; Mental Workout/Timeshifter and View Inc. He has received honoraria and travel or accommodation expenses from Emory University, Estée Lauder, Ineos, MIT, Roxbury Latin School, and University of Toronto, and travel or accommodation expenses (no honoraria) from IES, Mental Workout, Solemma, and Wiley; and royalties from Oxford University Press. He holds equity in iSleep pty. He has received an unrestricted equipment gift from F. Lux Software LLC, a fellowship gift from Stockgrand Ltd. and holds an investigator-initiated grant from F. Lux Software LLC and a Clinical Research Support Agreement with Vanda Pharmaceuticals Inc. He is an unpaid Board Member of the Midwest Lighting Institute (non-profit). He was a Program Leader for the CRC for Alertness, Safety and Productivity, Australia, through an adjunct professor position at Monash University (2015–2019). He has served as a paid expert in legal proceedings related to light, sleep and health. The remaining authors declare that the research was conducted in the absence of any commercial or financial relationships that could be construed as a potential conflict of interest.

## Publisher's Note

All claims expressed in this article are solely those of the authors and do not necessarily represent those of their affiliated organizations, or those of the publisher, the editors and the reviewers. Any product that may be evaluated in this article, or claim that may be made by its manufacturer, is not guaranteed or endorsed by the publisher.
