# Supplementary material for: Development of a Home-Based Light Therapy for Fatigue Following Traumatic Brain Injury: Two Case Studies
Source: Front Neurol. 2021 Sep 13;12:651498. doi: 10.3389/fneur.2021.651498 (PMC8473693; doi:10.3389/fneur.2021.651498)
Supplement: Supplementary file 1 [file Data_Sheet_1.docx]

Supplementary Material

**Supplemental Materials**

The primary outcome measure was the *Brief Fatigue Inventory (BFI)*, a 9-item scale used to capture current fatigue levels on a scale of 0 (no fatigue or does not currently interfere) to 10 (bad fatigue that completely interferes with activity/work) in the previous 24 hours (state fatigue). Scores 4-7 represent moderate fatigue and suggest a need for intervention, scores ≥ 8 represent severe fatigue.(1) It has been shown to be sensitive to treatment response in TBI (2).

Secondary Outcomes included the following measures.

*Fatigue Severity Scale (FSS)*(3), 9-item self-report measure assessing impact of fatigue on daily activities or trait-like fatigue, on a 7-point scale from 1 (strongly disagree) to 7 (strongly agree). A mean item score ≥ 4 indicates clinically significant fatigue.

*Epworth Sleepiness Scale (ESS)* includes 8 items assessing a person’s likelihood of falling asleep during everyday activities such as “Watching TV” or “Sitting quietly after a lunch without alcohol”. Score > 10 suggests clinically significant daytime sleepiness.(4)

*Pittsburgh Sleep Quality Index (PSQI)* assesses subjective global sleep quality(5) in past month (e.g. bedtime, sleep duration) and rates frequency of problems interfering with sleep. Lower scores indicate greater sleep quality. Scores ≥ 5 indicate clinically significant sleep disturbance.

*Insomnia Severity Index (ISI)*(6) screens for insomnia with 7 questions rated on a 5-point scale, (0 = no problem, 4 = very severe problem). A score of 8-14 indicates subthreshold insomnia, 15-21 clinically moderate, and 22 or greater severe clinical insomnia.

*Psychomotor Vigilance Task (PVT)* (10 min) was used to measure sustained attention and reaction time using a response box and testing computer. Prior research has demonstrated exposure to short wavelength light, decreases reaction time and errors on this task.(7)

*Hospital Anxiety and Depression Scale (HADS)* measures self-reported anxiety and depression symptoms(8) with anxiety (HADS-A) and depression (HADS-D) subscales. The 14 items are rated on a 4-point scale, where 0 = “Not at all” and 3 = “Most of the time”. Scores ≥ 8 on each subscale are considered to be indicative of clinically significant symptoms, with higher scores representing greater severity.

*Participation Objective Participation Subjective (POPS)*(9) assessed community and social participation by measuring frequency of productivity including work and study, ‘out and about’ and social relations, higher scores indicating greater participation.

*Actigraphy and Sleep Diary.* Participants recorded sleep and wake times and other sleep phenomena, in a daily sleep diary throughout the study, including time to fall asleep, awakenings after sleep onset, and daytime naps. They also wore wrist actigraphy devices (Actiwatch-2, Actiwatch Spectrum or Actiwatch Spectrum Plus; Philips Respironics, Bend, OR, USA). Actigraphy devices were worn on the non-dominant wrist, with activity measured in 1-minute epoch as sleep or wake. Actigraphic sleep parameter estimates were analyzed for the sleep episodes identified in the sleep diaries (Actiware 5 software, Philips Respironics Inc, Bend, OR, USA). Individual actigraphic sleep episodes were inspected and aligned with sleep diaries by inputting of subjective sleep and wake times by an independent researcher blinded to study conditions. When discrepancies ≥ 60 minutes between actigraphy data and sleep diary entries were identified, the following approach was used: If subjective bedtime was reported as ≥ 60 min before a substantial reduction in activity and light levels, bedtime was adjusted to the time of decreased activity and light; if reported wake time was ≥ 60 min after a substantial increase in activity and light, wake time was shifted to the start of the sustained activity and light increase. From this data the following six outcomes were derived, which were an average of an individual participant’s data across a given study period: total sleep time (TST), sleep onset latency, wake after sleep onset (WASO), sleep efficiency (%), as well as participants’ average sleep and wake times.

*Side Effects Questionnaire* was used to capture side effects experienced, including headache, nausea, changes in cognition, and appetite, at each assessment time-point.

*End of Light Therapy Questionnaire* was completed at follow-up, to capture participants’ qualitative experiences of the lighting interventions and subjective changes in symptoms.

**Supplementary Table 1. Case 1 Lighting Protocols.**

| **Location** |  | **Baseline**  **(pre-existing lighting)** | **Treatment** | **Control** |
| --- | --- | --- | --- | --- |
| Bedroom | Hardware | Pendant – 1 fluorescent globe, 4000K, 20W | Pendant – 1 x Philips Scene Switch, 3000K/6500K, 806lm, 9.5W | Pendant – 1 x GE LED GLS Bulb, 4000K, 820lm, 8.8W |
|  |  | Lamp – 1 x warm white halogen globe, 1150lm | Lamp – 1 x Lighting Science GoodNight LED bulb, 2175K, 600lm, 8.5W | Lamp – 1 x warm white halogen globe, 1150lm (baseline bulb) |
|  | Condition |  | Pendant  Day: 6500K  Night: 3000K (I/N)  Lamp – Night: on |  |
| Living | Hardware | 2 x “Cool Daylight” 1040lm, 18W | 2 x Philips Scene Switch, 3000K/6500K, 806lm, 9.5W | 2 x GE LED GLS Bulb, 4000K, 820lm, 8.8W |
|  | Condition |  | Day: 6500K  Night: 3000K (I/N) |  |
| Kitchen | Hardware | 1 x fluorescent, 2700K, 15W | 1 x Philips Scene Switch, 3000K/6500K, 806lm, 9.5W | 1 x Philips, 2700K, 806lm, 7W |
|  | Condition |  | Day: 6500K  Night: 3000K (I/N) |  |
| Study | Hardware | 1 x halogen globe, 72W | 1 x Philips Scene Switch, 3000K/6500K, 806lm, 9.5W  Lamp provided with 1 x Philips Scene Switch, 3000K/6500K, 806lm, 9.5W | 1 x Philips LED Bulb, 3000K, 806lm, 8W |
|  | Condition |  | Day: 6500K  Night: 3000K (I/N) |  |
| Bathroom | Hardware | 1 x 601lm, 42W | 1 x Philips Scene Switch 3000K/6500K, 806lm, 9.5W | 1 x Osram LED, 2700K, 470lm, 4.5W |
|  | Condition |  | Day: 6500K  Night: 3000K (I/N) |  |
| Devices | Laptop, phone | No f.lux | f.lux set to ON | f.lux set to OFF |

*Notes.* DL = down lights, lm = lumens, K = Kelvin, CCT = correlated color temperature, W = Watts, I/N = if needed.

**Supplementary Table 2. Case 1 Baseline Lighting Measurements.**

| **Location (Time)** | **Height, plane** | **Pos.** | **Photopic lux** | **CCT (K)** | **Irradiance uW/cm^2^** | **S-cone-opic a-opic EDI (lux)** | **M-cone-opic a-opic EDI (lux)** | **L-cone-opic a-opic EDI (lux)** | **Rhodopic a-opic EDI (lux)** | **Melanopic a-opic EDI (lux)** | **Melanopic DER** |
| --- | --- | --- | --- | --- | --- | --- | --- | --- | --- | --- | --- |
|  |  |  | **Mean ±**  **SD** | **Mean ±**  **SD** | **Mean ±**  **SD** | **Mean ±**  **SD** | **Mean ±**  **SD** | **Mean ±**  **SD** | **Mean ±**  **SD** | **Mean ±**  **SD** | **Mean ±**  **SD** |
| Living area (Day) | 52", vert x 4 | A | 160.22 ± 76.63 | 4905 ±  148 | 64.29 ± 34.30 | 120.23 ± 62.04 | 150.63 ± 74.17 | 159.85 ± 76.84 | 135.13 ± 70.71 | 129.30 ± 69.68 | 0.79 ±  0.04 |
|  | 72", horiz | A | 458.24 | 5661 | 162.61 | 389.09 | 437.89 | 451.53 | 390.26 | 368.62 | 0.80 |
| Living area (Evening) | 52", vert x 4 | A | 45.18 ± 24.41 | 5201 ±  339 | 15.30 ± 8.41 | 36.41 ± 21.61 | 42.53 ± 23.41 | 44.56 ± 24.03 | 37.09 ± 20.98 | 34.67 ± 19.83 | 0.76 ±  0.03 |
|  | 72", horiz | A | 335.77 | 5928 | 111.90 | 299.25 | 322.31 | 329.67 | 287.24 | 270.33 | 0.81 |
| Kitchen (Day) | 52", vert x 4 | B | 280.27 ± 188.70 | 4506 ±  875 | 118.77 ± 88.33 | 221.99 ± 192.82 | 262.33 ± 188.57 | 281.63 ± 189.15 | 239.33 ± 189.38 | 231.84 ± 190.18 | 0.77 ±  0.13 |
|  | 72", horiz | B | 293.58 | 3089 | 98.95 | 119.08 | 239.69 | 296.33 | 167.52 | 142.32 | 0.48 |
| Kitchen (Evening) | 52", vert x 4 | B | 162.67 ± 163.86 | 3823 ±  890 | 66.89 ± 72.63 | 115.31 ± 143.67 | 148.65 ± 158.89 | 163.75 ± 164.12 | 130.81 ± 151.94 | 124.84 ± 149.59 | 0.66 ±  0.15 |
|  | 72", horiz | B | 267.21 | 2904 | 87.36 | 97.96 | 213.35 | 270.54 | 143.10 | 118.80 | 0.44 |
| Bathroom (Day) | 52", vert x 4 | C | 241.93 ± 218.56 | 4053 ±  852 | 111.29 ± 97.70 | 171.09 ± 199.24 | 223.76 ± 215.29 | 243.26 ± 218.39 | 200.43 ± 209.30 | 192.65 ± 207.07 | 0.72 ±  0.11 |
|  | 72", horiz | C | 220.83 | 3186 | 107.23 | 92.52 | 185.18 | 223.93 | 141.80 | 127.62 | 0.58 |
| Bathroom (Evening) | 52", vert x 4 | C | No blinds in bathroom.^a^ | | |  |  |  |  |  |  |
|  | 72", horiz | C |  |  |  |  |  |  |  |  |  |
| Study  (Day) | 52", vert x 4 | E | 217.05 ± 189.50 | 4448 ±  834 | 96.61 ± 82.33 | 164.48 ± 168.97 | 204.24 ± 186.06 | 217.77 ± 189.27 | 187.04 ± 179.87 | 181.23 ± 177.53 | 0.78 ±  0.11 |
|  | 72", horiz | E | 334.01 | 3364 | 159.86 | 157.05 | 284.91 | 338.20 | 224.91 | 205.29 | 0.61 |
| Study (Evening) | 52", vert x 4 | E | 36.73 ± 32.22 | 2579 ±  72 | 19.50 ± 16.39 | 9.29 ± 8.16 | 28.56 ± 25.27 | 37.56 ± 32.88 | 19.10 ± 17.00 | 16.10 ± 14.34 | 0.44 ±  0.02 |
|  | 72", horiz | E | 228.83 | 2649 | 117.85 | 57.70 | 179.22 | 233.51 | 120.33 | 101.33 | 0.44 |
| Bedroom (Day) | 52", vert x 4 | F | 201.44 ± 157.11 | 4555 ±  702 | 82.61 ± 69.23 | 152.00 ± 143.12 | 189.18 ± 154.81 | 201.61 ± 157.08 | 171.18 ± 151.00 | 164.73 ± 149.73 | 0.76 ±  0.11 |
|  | 72", horiz | F | 361.71 | 3974 | 120.11 | 200.80 | 317.75 | 359.74 | 246.85 | 219.25 | 0.61 |
| Bedroom (Evening) | 52", vert x 4 | F | 33.72 ±  5.26 | 3512 ±  164 | 10.60 ± 1.54 | 15.29 ± 2.64 | 28.54 ±  4.49 | 33.66 ± 5.21 | 20.70 ± 3.29 | 17.73 ± 2.87 | 0.53 ±  0.04 |
|  | 72", horiz | F | 354.60 | 3742 | 106.30 | 173.73 | 304.05 | 351.58 | 220.59 | 187.50 | 0.53 |

*Notes*. Abbreviations: CCT (correlated color temperature); EDI (Equivalent Daylight Illuminance; DER (Daylight Equivalent Ratio). Only the melDER is shown here; DER values for the other photoreceptors can be calculated by dividing the α-opic EDI value by the photopic lux provided.

^a^As evening light measurements were taken with window blinds closed, to approximate night time lighting conditions, measurements were not possible in locations without blinds.

**Supplementary Table 3**

*Case 1 Treatment and Control Condition Supplementary Lighting Measurements*

| **Location (Time)** | **Height, plane** | **Pos.** | **Irradiance uW/cm^2^**  **TREAT.** | **Irradiance uW/cm^2^**  **CONTROL** | **S-cone-opic a-opic EDI (lux)**  **TREAT.** | **S-cone-opic a-opic EDI (lux)**  **CONTROL** | **M-cone-opic a-opic EDI (lux)**  **TREAT.** | **M-cone-opic a-opic EDI (lux)**  **CONTROL** | **L-cone-opic a-opic EDI (lux)**  **TREAT.** | **L-cone-opic a-opic EDI (lux)**  **CONTROL** | **Rhodopic a-opic EDI (lux)**  **TREAT.** | **Rhodopic a-opic EDI (lux)**  **CONTROL** |
| --- | --- | --- | --- | --- | --- | --- | --- | --- | --- | --- | --- | --- |
|  |  |  | **Mean ±**  **SD** | **Mean ±**  **SD** | **Mean ±**  **SD** | **Mean ±**  **SD** | **Mean ±**  **SD** | **Mean ±**  **SD** | **Mean ±**  **SD** | **Mean ±**  **SD** | **Mean ±**  **SD** | **Mean ±**  **SD** |
| Living area (Day) | 52", vert x 4 | A | 35.89 ± 21.29 | 52.62 ± 34.54 | 88.88 ± 63.74 | 99.77 ± 78.06 | 97.37 ±  65.41 | 121.54 ± 75.35 | 100.44 ± 66.24 | 130.61 ± 74.95 | 90.50 ± 61.3 | 109.74 ± 76.52 |
|  | 72", horiz | A |  | 102.12 |  | 174.65 |  | 269.24 |  | 303.74 |  | 217.20 |
| Living area (Evening) | 52", vert x 4 | A | 9.08 ±  6.69 | 11.98 ± 7.01 | 9.59 ±  7.82 | 18.69 ± 12.74 | 22.65 ±  17.22 | 30.42 ± 18.34 | 28.78 ± 21.44 | 34.98 ± 20.47 | 14.99 ± 11.72 | 24.02 ± 15.16 |
|  | 72", horiz | A | 51.39 | 57.71 | 57.69 | 96.36 | 134.80 | 161.94 | 168.96 | 186.17 | 89.63 | 124.63 |
| Kitchen (Day) | 52", vert x 4 | B | 41.56 ± 36.23 | 86.03 ± 94.47 | 89.07 ± 73.44 | 192.00 ± 251.75 | 99.99 ±  77.42 | 187.09 ± 199.98 | 103.95 ± 78.52 | 194.07 ± 190.64 | 93.70 ± 76.27 | 183.16 ± 217.39 |
|  | 72", horiz | B | 63.19 | 100.74 | 162.65 | 119.79 | 176.08 | 237.65 | 181.2 | 293.92 | 163.97 | 170.47 |
| Kitchen (Evening) | 52", vert x 4 | B | 25.87 ± 18.71 | 57.19 ± 56.00 | 38.83 ± 39.86 | 124.16 ± 158.29 | 60.07 ±  40.11 | 124.78 ± 116.10 | 69.75 ± 40.18 | 132.25 ± 108.17 | 48.16 ± 40.36 | 119.22 ± 130.71 |
|  | 72", horiz | B | 55.11 | 113.69 | 65.75 | 113.09 | 140.54 | 273.47 | 172.69 | 348.96 | 97.94 | 182.39 |
| Bathroom (Day) | 52", vert x 4 | C | 54.34 ± 41.11 | 93.00 ± 64.32 | 116.27 ± 93.60 | 205.26 ± 174.71 | 139.83 ± 101.32 | 199.60 ± 138.58 | 146.65 ± 103.11 | 204.37 ± 131.48 | 125.92 ± 96.25 | 197.79 ± 150.76 |
|  | 72", horiz | C | 108.96 | 84.66 | 278.35 | 106.51 | 318.11 | 191.91 | 330.46 | 229.21 | 284.59 | 147.26 |
| Bathroom (Evening) | 52", vert x 4 | C | No blinds in bathroom^a^ | |  |  |  |  |  |  |  |  |
|  | 72", horiz | C |  |  |  |  |  |  |  |  |  |  |
| Study  (Day) | 52", vert x 4 | E | 41.56 ± 27.72 | 60.40 ± 60.78 | 99.21 ± 67.47 | 120.35 ± 148.91 | 113.09 ± 72.36 | 138.29 ± 136.91 | 117.43 ± 73.39 | 146.10 ± 133.51 | 103.24 ± 68.73 | 127.44 ± 140.15 |
|  | 72", horiz | E | 114.78 | 145.35 | 311.96 | 193.84 | 341.31 | 369.69 | 350.93 | 445 | 312.32 | 273.15 |
| Study  (Evening) | 52", vert x 4 | E | 22.21 ± 11.12 | 8.36 ±  1.51 | 22.45 ± 12.26 | 8.24 ±  1.82 | 57.37 ±  29.34 | 21.37 ±  3.90 | 73.15 ± 36.70 | 27.49 ±  4.79 | 37.10 ± 19.47 | 14.00 ±  2.75 |
|  | 72", horiz | E | 67.54 | 87.85 | 71.30 | 98.90 | 177.06 | 232.81 | 223.64 | 292.54 | 115.66 | 157.45 |
| Bedroom (Day) | 52", vert x 4 | F | 26.20 ± 19.46 | 100.61 ± 74.33 | 58.91 ± 42.18 | 219.57 ± 186.14 | 66.12 ±  43.39 | 229.99 ± 166.73 | 68.53 ± 43.53 | 236.00 ± 161.84 | 61.44 ± 43.18 | 222.41 ± 173.89 |
|  | 72", horiz | F | 87.02 | 129.11 | 239.91 | 241.13 | 255.49 | 326.85 | 261.99 | 356.01 | 234.96 | 282.00 |
| Bedroom (Evening) | 52", vert x 4 | F | 7.40 ±  2.00 | 26.34 ± 16.08 | 6.89 ±  1.95 | 20.52 ±  6.30 | 18.39 ±  5.20 | 48.52 ±  22.74 | 23.73 ±  6.68 | 60.91 ± 30.87 | 11.68 ±  3.30 | 34.01 ± 14.27 |
|  | 72", horiz | F | 73.71 | 76.12 | 80.30 | 103.50 | 193.64 | 191.05 | 243.47 | 223.86 | 126.94 | 143.73 |
| Bedroom (Evening; lamp only)^b^ | 52", vert x 4 | F | 8.91 ±  8.12 |  | 2.20 ±  1.50 |  | 15.82 ±  14.83 |  | 23.09 ± 21.69 |  | 8.63 ±  7.89 |  |
|  | 72", horiz | F | 11.22 |  | 3.02 |  | 20.25 |  | 29.34 |  | 11.14 |  |

*Notes*. Abbreviations: CCT (correlated color temperature); EDI (Equivalent Daylight Illuminance; DER (Daylight Equivalent Ratio). DER values for the other photoreceptors can be calculated by dividing the α-opic EDI value by the photopic lux provided in Table 1.

The aim of the intervention was to increase the melEDI and DER during the day and decrease them during the evening during the Treatment compared to the Control intervention (final two columns). This was typically associated with a daytime increase and evening decrease in CCT during the Treatment.

Average calculations found in text generated using 72” measurements.

^a^As evening light measurements were taken with window blinds closed, to approximate night time lighting conditions, measurements were not possible in locations without blinds.

^b^An additional measure that was not captured in the Control condition.

**Supplementary Table 4. Case 2 Lighting Protocols.**

| **Location** |  | **Baseline (pre-existing lighting)** | **Treatment** | **Control** |
| --- | --- | --- | --- | --- |
| Bedroom | Hardware | Ceiling – 1 x “Natural White”, 15W  Lamp – 1 x 6500K, 470lm, 5W | Ceiling - 1 x Lighting Science GoodNight LED bulb, 2175K, 600lm, 8.5W, M/P 0.33  Lamp – replaced with 1 x Lighting Science Genesis DynaSpectrum HealthE LED lamp (circadian mode), 2200-7000K, 450lm | Ceiling - 1 x “Natural White”, 15W (baseline light)  Lamp – 1 x 6500K, 470lm, 5W (baseline light) |
|  | Condition |  | Ceiling  Day: ON as needed  Night: ON (preference for lamp only)  Lamp  Day: ON  Night: ON (I/N) |  |
| Living | Hardware | Ceiling – 1 x “Natural White”, 15W  Lamp - 1 x ~4000-5000K, 60W | Ceiling - 1 x Philips Scene Switch 3000K/6500K, 806lm, 9.5W  Lamp - 1 x Lighting Science GoodNight LED bulb, 2175K, 600lm, 8.5W | Ceiling – 1 x “Natural White”, 15W (baseline light)  Lamp - 1 x ~4000-5000K, 60W (baseline light) |
|  | Condition |  | Ceiling  Day: 6500K ON  Night: 3000K ON if needed  Lamp  Day: OFF  Night: ON |  |
| Kitchen | Hardware | 1 x 120cm T8 fluorescent tube light, ~4000K | 1 x 120cm T8 fluorescent tube light, ~4000K (baseline light)  Lamp provided with 1 x Lighting Science GoodDay LED bulb, 5000K, 800lm, 9W | 1 x Osram 120cm T8 fluorescent tube, 3000K, 3350lm, 36W |
|  | Condition |  | Ceiling  Day: ON  Night: OFF  Lamp  Day: ON  Night: OFF |  |
| Bathroom | Hardware | 1 x 60cm T8 fluorescent tube light, ~4000K | 1 x Osram 60cm T8 fluorescent tube, 3000K, 1350lm, 18W | 1 x 60cm T8 fluorescent tube, ~4000K (baseline light) |
|  | Condition |  | Day: ON as needed  Night: ON as needed |  |
| Devices | iPad | No Night Shift setting used | Night Shift set to ON | Night Shift set to OFF |

*Notes.* DL = down lights, lm = lumens, K = Kelvin, CCT = correlated color temperature, W = Watts, I/N = if needed.

**Supplementary Table 5. Case 2 Baseline Lighting Measurements.**

| **Location**  **(Time)** | **Height, plane** | **Pos.** | **Photopic lux** | **CCT (K)** | **Irradiance uW/cm^2^** | **S-cone-opic EDI (lux)** | **M-cone-opic EDI (lux)** | **L-cone-opic EDI (lux)** | **Rhodopic EDI (lux)** | **Melanopic EDI (lux)** | **Melanopic DER** |
| --- | --- | --- | --- | --- | --- | --- | --- | --- | --- | --- | --- |
|  |  |  | **Mean ±**  **SD** | **Mean ± SD** | **Mean ±**  **SD** | **Mean ±**  **SD** | **Mean ±**  **SD** | **Mean ±**  **SD** | **Mean ±**  **SD** | **Mean ±**  **SD** | **Mean ±**  **SD** |
| Bedroom (Day) | 52", vert x 4 | A | 34.10 ± 27.12 | 3626 ± 402 | 16.02 ±  11.91 | 13.95 ± 13.54 | 29.89 ± 24.79 | 33.82 ± 26.68 | 22.59 ± 19.77 | 19.83 ± 17.73 | 0.55 ±  0.05 |
|  | 72", horiz | A | 114.23 | 2500 | 62.19 | 23.21 | 87.20 | 116.80 | 55.36 | 45.19 | 0.40 |
| Bedroom (Evening) | 52", vert x 4 | A | 10.30 ±  3.77 | 2475 ± 42 | 6.06 ±  2.07 | 1.80 ±  0.60 | 7.79 ±  2.84 | 10.51 ±  3.86 | 4.76 ±  1.73 | 3.79 ±  1.38 | 0.37 ±  0.00 |
|  | 72", horiz | A | 82.92 | 2371 | 46.48 | 14.55 | 61.94 | 85.14 | 38.02 | 30.54 | 0.37 |
| Living (Day) | 52", vert x 4 | B | 64.67 ± 66.37 | 4210 ± 216 | 27.27 ±  28.47 | 32.08 ± 32.09 | 58.63 ± 60.69 | 63.78 ± 65.23 | 46.44 ± 48.15 | 41.61 ± 43.04 | 0.63 ±  0.05 |
|  | 72", horiz | B | 37.74 | 3985 | 13.11 | 17.60 | 33.20 | 37.27 | 24.96 | 21.69 | 0.57 |
| Living (Evening) | 52", vert x 4 | B | 14.99 ±  9.07 | 3787 ± 171 | 4.40 ±  2.69 | 6.92 ±  4.69 | 12.86 ±  7.79 | 14.82 ±  9.00 | 8.79 ±  5.36 | 7.45 ±  4.64 | 0.49 ±  0.03 |
|  | 72", horiz | B | 46.39 | 4080 | 13.10 | 21.54 | 40.69 | 45.47 | 29.30 | 24.70 | 0.53 |
| Kitchen (Day) | 52", vert x 4 | C | 184.96 ± 97.99 | 4162 ± 303 | 72.07 ±  41.72 | 101.68 ± 57.92 | 166.33 ± 90.41 | 183.32 ± 96.75 | 130.41 ± 74.65 | 118.01 ± 68.57 | 0.62 ±  0.06 |
|  | 72", horiz | C | 230.94 | 3898 | 77.43 | 118.43 | 200.89 | 229.71 | 145.56 | 128.72 | 0.56 |
| Kitchen (Evening) | 52", vert x 4 | C | 63.87 ± 14.84 | 3884 ± 108 | 19.29 ±  4.52 | 33.05 ±  8.03 | 55.04 ± 12.94 | 63.33 ± 14.63 | 37.88 ± 8.96 | 32.80 ± 7.74 | 0.51 ±  0.02 |
|  | 72", horiz | C | 694.33 | 3944 | 205.43 | 377.69 | 599.23 | 688.59 | 412.26 | 358.27 | 0.52 |
| Bathroom (Day) | 52", vert x 4 | D | 22.80 ± 26.65 | 4841 ± 604 | 10.11 ±  11.79 | 17.54 ± 23.06 | 21.94 ± 26.33 | 22.69 ± 26.48 | 20.27 ± 25.24 | 19.62 ± 24.74 | 0.80 ±  0.07 |
|  | 72", horiz | D | 15.90 | 4642 | 7.62 | 10.31 | 14.75 | 15.81 | 12.72 | 11.95 | 0.75 |
| Bathroom (Evening) | 52", vert x 4 | D | No blinds in bathroom.^a^ | | |  |  |  |  |  |  |
|  | 72", horiz | D |  |  |  |  |  |  |  |  |  |

*Notes*. Abbreviations: CCT (correlated color temperature); EDI (Equivalent Daylight Illuminance; DER (Daylight Equivalent Ratio); Pos. (Position). Only the melDER is shown here; DER values for the other photoreceptors can be calculated by dividing the α-opic EDI value by the photopic lux provided.

^a^As evening light measurements were taken with window blinds closed, to approximate night time lighting conditions, measurements were not possible in locations without blinds.

**Supplementary Table 6**

*Case 2 Treatment and Control Condition Supplementary Lighting Measurements*

| **Location (Time)** | **Height, plane** | **Pos.** | **Irradiance uW/cm^2^**  **TREAT.** | **Irradiance uW/cm^2^**  **CONTROL** | **S-cone-opic a-opic EDI (lux)**  **TREAT.** | **S-cone-opic a-opic EDI (lux)**  **CONTROL** | **M-cone-opic a-opic EDI (lux)**  **TREAT.** | **M-cone-opic a-opic EDI (lux)**  **CONTROL** | **L-cone-opic a-opic EDI (lux)**  **TREAT.** | **L-cone-opic a-opic EDI (lux)**  **CONTROL** | **Rhodopic a-opic EDI (lux)**  **TREAT.** | **Rhodopic a-opic EDI (lux)**  **CONTROL** |
| --- | --- | --- | --- | --- | --- | --- | --- | --- | --- | --- | --- | --- |
|  |  |  | **Mean ±**  **SD** | **Mean ±**  **SD** | **Mean ±**  **SD** | **Mean ±**  **SD** | **Mean ±**  **SD** | **Mean ±**  **SD** | **Mean ±**  **SD** | **Mean ±**  **SD** | **Mean ±**  **SD** | **Mean ±**  **SD** |
| Bedroom (Day) | 52", vert x 4 | A | 10.69 ± 2.74 | 16.02 ± 11.91 | 13.24 ± 7.40 | 13.95 ± 13.54 | 23.66 ± 8.01 | 29.89 ± 24.79 | 27.78 ±  8.00 | 33.82 ± 26.68 | 20.07 ± 9.33 | 22.59 ± 19.77 |
|  | 72", horiz | A | 41.40 | 62.19 | 12.13 | 23.21 | 75.69 | 87.20 | 110.40 | 116.80 | 41.95 | 55.36 |
| Bedroom (Evening) | 52", vert x 4 | A | 4.78 ±  1.02 | 8.14 ±  4.79 | 0.84 ±  0.16 | 16.95 ± 12.05 | 8.06 ±  1.76 | 25.01 ± 14.93 | 12.12 ±  2.55 | 26.83 ± 15.52 | 3.98 ±  0.91 | 19.86 ± 12.79 |
|  | 72", horiz | A | 42.04 | 30.06 | 6.48 | 57.54 | 73.84 | 90.85 | 111.58 | 99.28 | 36.95 | 68.78 |
| Living  (Day) | 52", vert x 4 | B | 5.38 ±  3.28 | 27.27 ± 28.47 | 9.30 ±  6.88 | 32.08 ± 32.09 | 14.06 ± 9.73 | 58.63 ± 60.69 | 15.12 ± 10.20 | 63.78 ± 65.23 | 11.48 ± 8.29 | 46.44 ± 48.15 |
|  | 72", horiz | B | 12.94 | 13.11 | 27.66 | 17.60 | 39.60 | 33.20 | 41.98 | 37.27 | 33.22 | 24.96 |
| Living (Evening) | 52", vert x 4 | B | 3.87 ±  2.54^b^ | 28.88 ± 41.76 | 7.67 ±  5.45^b^ | 59.84 ± 92.15 | 11.23 ± 7.76^b^ | 79.40 ± 116.65 | 12.19 ±  8.25^b^ | 84.44 ± 121.75 | 9.21 ±  6.53^b^ | 72.29 ± 108.88 |
|  | 72", horiz | B | 7.63 | 9.15 | 16.03 | 12.58 | 23.49 | 23.93 | 25.09 | 28.42 | 19.50 | 18.18 |
| Kitchen  (Day) | 52", vert x 4 | C | 40.56 ± 33.27 | 72.07 ± 41.72 | 77.55 ± 66.43 | 101.68 ± 57.92 | 108.67 ± 91.36 | 166.33 ± 90.41 | 117.68 ± 98.78 | 183.32 ± 96.75 | 91.49 ± 77.13 | 130.41 ± 74.65 |
|  | 72", horiz | C | 27.69 | 77.43 | 48.12 | 118.43 | 74.63 | 200.89 | 83.10 | 229.71 | 58.60 | 145.56 |
| Kitchen (Evening) | 52", vert x 4 | C | 20.53 ± 22.32^c^ | 14.64 ± 14.43 | 39.55 ± 45.11^c^ | 25.41 ± 26.73 | 56.93 ± 63.64^c^ | 42.66 ± 42.97 | 62.61 ± 69.80^c^ | 49.15 ± 48.81 | 45.97 ± 51.4^c^ | 29.27 ± 29.17 |
|  | 72", horiz | C | 11.78 | 18.73 | 20.80 | 32.62 | 32.40 | 55.19 | 36.09 | 63.49 | 26.18 | 37.51 |
| Bathroom (Day) | 52", vert x 4 | D | 12.47 ± 4.85 | 10.11 ± 11.79 | 13.47 ± 6.14 | 17.54 ± 23.06 | 31.95 ± 13.07 | 21.94 ± 26.33 | 40.41 ± 16.88 | 22.69 ± 26.48 | 21.2 ±  8.67 | 20.27 ± 25.24 |
|  | 72", horiz | D | 55.40 | 7.62 | 59.34 | 10.31 | 150.75 | 14.75 | 193.32 | 15.81 | 95.28 | 12.72 |
| Bathroom (Evening) | 52", vert x 4 | D | No blinds in bathroom.^a^ | 16.55 ± 10.16^d^ | No blinds in bathroom.^a^ | 17.41 ± 11.19^d^ | No blinds in bathroom.^a^ | 44.26 ± 27.84^d^ | No blinds in bathroom.^a^ | 57.41 ± 35.79^d^ | No blinds in bathroom.^a^ | 27.57 ± 17.38^d^ |
|  | 72", horiz | D |  | 51.00 |  | 56.27 |  | 138.42 |  | 177.84 |  | 86.97 |

*Notes*. Abbreviations: CCT (correlated color temperature); EDI (Equivalent Daylight Illuminance; DER (Daylight Equivalent Ratio); Pos. (Position). DER values for the photoreceptors, can be calculated by dividing the α-opic EDI value by the photopic lux provided, found in Table 3.

The aim of the intervention was to increase the melEDI and DER during the day and decrease them during the evening during the Treatment compared to the Control intervention. This was typically associated with a daytime increase and evening decrease in CCT during the Treatment.

Average calculations found in text generated using 72” measurements. Bedroom and bathroom measurements were excluded from Treatment ‘day’ average calculations as these spaces were used exclusively during evenings and daytime lighting was not fitted. The following measures were excluded from Treatment ‘evening’ calculation: Kitchen measurement excluded as this space was used exclusively during daytime and evening lighting was not fitted; Living was excluded from ‘evening’ calculation as daytime lighting was primarily measured, as noted below; Bathroom was excluded as no blinds were available to block natural light from windows in this space.

Bedroom and bathroom measurements were excluded from Control ‘day’ average calculations as these spaces were used exclusively during evenings and daytime lighting was not fitted during Treatment. The following measures were excluded from Control ‘evening’ calculation: Kitchen measurement excluded as this space was used exclusively during daytime and evening lighting was not fitted during Treatment; Living was excluded from ‘evening’ calculation as daytime lighting was primarily measured, as noted below; Bathroom was excluded as no blinds were available to block natural light from windows in this space.

^a^As evening light measurements were taken with window blinds closed, to approximate night time lighting conditions, measurements were not possible in locations without blinds, if assessed during daytime.

^b^Lighting Science GoodNight light (2175K, 600lm) used in a lamp in this space for evening use, with an established M/P of 0.33. It is likely light was blended with overhead daytime lighting at the time of measurement due to researcher error.

^c^Single spectrum high melanopic light only was installed in the kitchen, based on exclusive daytime use. Evening measures are therefore not reflective of actual night time exposure in this location.

^d^Measurement was captured in evening, after sunset.

**Supplementary Figure 1A.** Case Study 1 Floor Plan (one level, ground floor). *Notes*. Letters represent positions at which light measurements were taken. Wall dimensions in metres. Floor plans designed in SmartDraw software.(10)

*
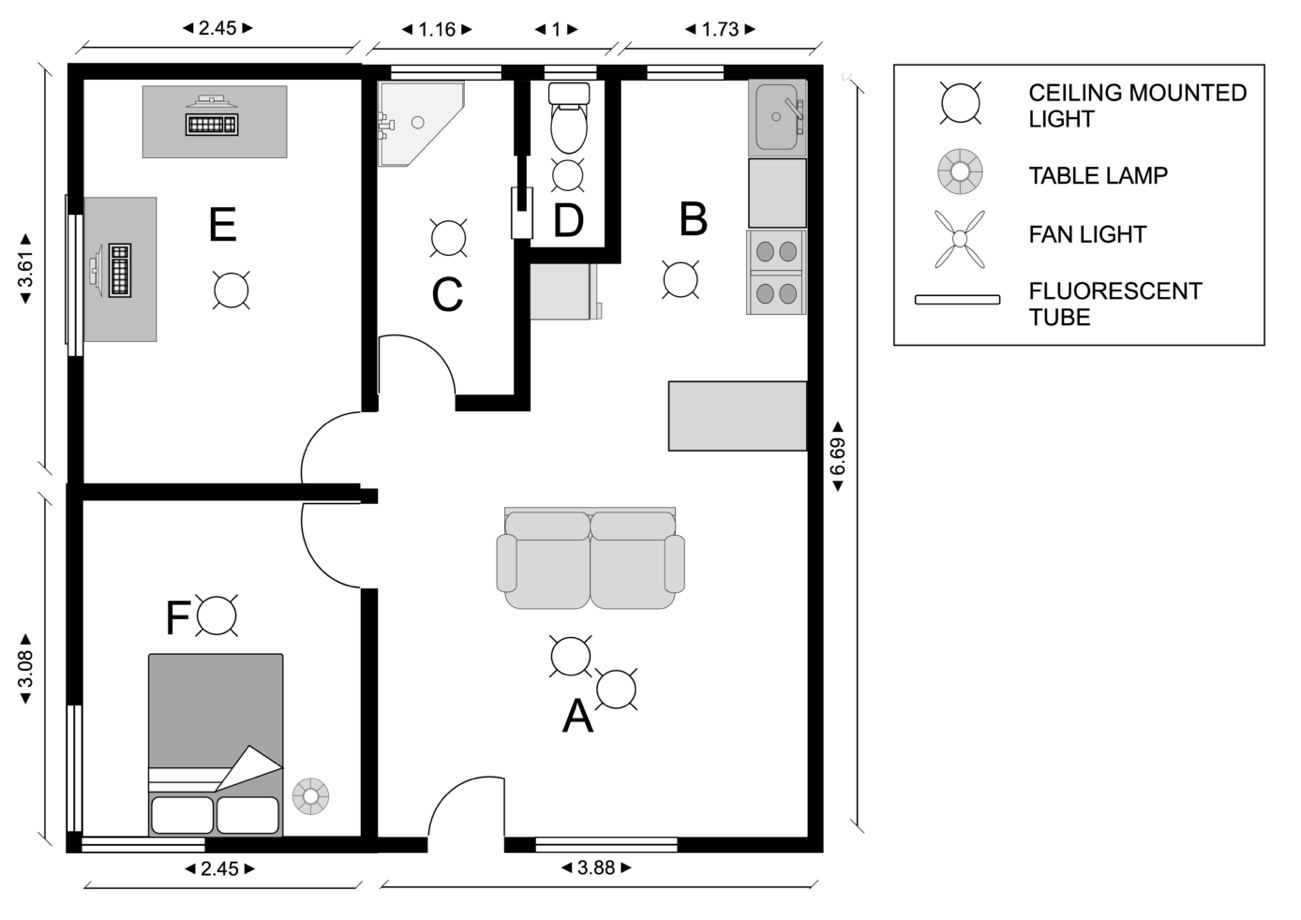
*

**Supplementary Figure 1B.** Case Study 2 Floor Plan (one level, ground floor).


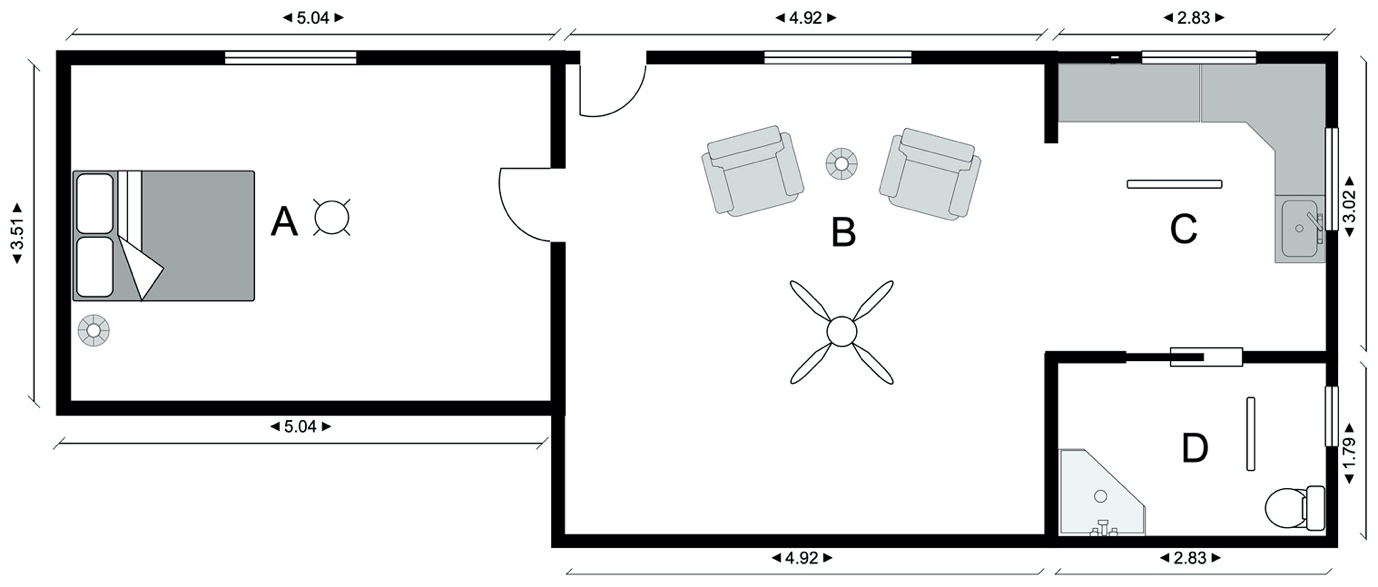


**References**

1. Chang YJ, Lee JS, Lee CG, Lee WS, Lee KS, Bang S-M, et al. Assessment of clinical relevant fatigue level in cancer. Support Care Cancer. 2007;15(7):891-6.

2. Sinclair KL, Ponsford JL, Taffe J, Lockley SW, Rajaratnam SM. Randomized controlled trial of light therapy for fatigue following traumatic brain injury. Neurorehabil Neural Repair. 2014;28(4):303-13.

3. Krupp LB, LaRocca NG, Muir-Nash J, Steinberg AD. The fatigue severity scale. Application to patients with multiple sclerosis and systemic lupus erythematosus. Arch Neurol. 1989;46(10):1121-3.

4. Johns MW. A new method for measuring daytime sleepiness: The Epworth sleepiness scale. Sleep. 1991;14(6):540-5.

5. Buysse DJ, Reynolds CF, 3rd, Monk TH, Berman SR, Kupfer DJ. The Pittsburgh Sleep Quality Index: A new instrument for psychiatric practice and research. Psychiatry Res. 1989;28(2):193-213.

6. Bastien CH, Vallieres A, Morin CM. Validation of the Insomnia Severity Index as an outcome measure for insomnia research. Sleep Med. 2001;2(4):297-307.

7. Lockley SW, Evans EE, Scheer FA, Brainard GC, Czeisler CA, Aeschbach D. Short-wavelength sensitivity for the direct effects of light on alertness, vigilance, and the waking electroencephalogram in humans. Sleep. 2006;29(2):161-8.

8. Zigmond AS, Snaith RP. The Hospital Anxiety and Depression Scale. Acta Psychiatr Scand. 1983;67(6):361-70.

9. Brown M, Dijkers MP, Gordon WA, Ashman T, Charatz H, Cheng Z. Participation Objective, Participation Subjective: A measure of participation combining outsider and insider perspectives. J Head Trauma Rehabil. 2004;19(6):459-81.

10. Smart Draw LLC. SmartDraw 2020 [Software]. Available from: <https://cloud.smartdraw.com/>.
